# Supplementary material for: Are carbon emissions trading and green financial instruments synergistic? -Comprehensive quantitative research based on content analysis
Source: PLoS One. 2024 Mar 7;19(3):e0298601. doi: 10.1371/journal.pone.0298601 (PMC10919601; doi:10.1371/journal.pone.0298601)
Supplement: S1 Data — (ZIP) [file pone.0298601.s001.zip › Data/地级市Z_F工作报告.zip!/║┌┴·╜¡/╥┴┤║╩╨/└┤╫╘╣1⁄2╓┌║┼í╛┬φ┐╦╩2╛▌═°í┐_2012.docx]

各位代表：

现在，我代表市政府向大会作工作报告，请予审议，并请市政协委员和列席会议的同志提出意见。

一、过去五年工作回顾

过去的五年，是伊春发展史上浓墨重彩的五年。五年来，在省委、省政府和市委的正确领导下，市政府团结带领全市人民，全面贯彻落实科学发展观，抢抓机遇，攻坚克难，圆满完成了市十二届人大各次会议确定的目标任务。

——过去五年，我们集中精力抓发展，经济增长的内生动力显著增强。果断调整发展思路，大力实施“产业兴市”战略，通过转方式、调结构、上项目，接续替代产业不断壮大，发展基础日益夯实。2011年，全市地区生产总值预计完成230亿元，比2006年增长77.1%。全口径财政收入由6亿元猛增到24.5亿元，翻了两番，占GDP的比重由5.3%提高到10.6%。固定资产五年累计投资535亿元，是前五年总和的4.5倍。森林食品业增加值实现倍增。风电总装机容量达到37万千瓦，增长6.3倍。钢铁、水泥产能双双跃上300万吨台阶，分别增长1.3倍和3.3倍。储量达89万吨的钼矿发现并开工建设。旅游收入突破35亿元，增长近3倍。这些数据表明，我们的快速增长不是单项的突破，而是多点支撑，均衡、协调、可持续的发展。

——过去五年，我们持之以恒保生态，森林资源得到明显恢复和发展。抓住我省建设“八大经济区”的历史机遇，创造性实施天保工程，通过严管资源、保护珍贵物种、大力营造林、实施生态移民和烧柴改革、强化森防能力建设等举措，系统化推进生态功能区建设，全市森林覆盖率由80.6%提高到83.9%，森林蓄积由2.35亿立方米增加到2.56亿立方米，林木年净增长量由500万立方米提高到700万立方米以上。通过深入开展保护红松行动、定期发布年度生态报告、开展森林湿地生态价值评估、创办东北亚生态论坛等活动，我市保护生态的理念和做法在全国乃至全球产生了较大影响。以停伐为标志，我市进入了森林经营新时代，“青山常在、永续利用”、“越采越多、越采越好”的理想和追求正在加速成为现实。

——过去五年，我们创新机制增活力，改革开放日益深化。国有林权制度改革稳步推进，综合效应日益显现。西钢、浩水、光明、啤酒厂重组改制，水解厂等一批停产半停产企业成功回购，闲置多年的资产得以盘活。投融资体制改革取得实质性进展，龙江银行挂牌运营，农村信用社改制顺利推进。土地管理制度不断完善，市本级土地出让金累计收益12.6亿元。公共资源交易管理实现突破，在全省率先建立统一交易平台，对政府公共权益与服务实施了公开监管。共建机制不断创新，铁力区域“1+5”、嘉荫场县共建模式极大地推动了新农村、新林区协调发展。对内对外务实合作成效显著，宝宇、龙源、通德、中铁、华润、华能等一大批战略投资者相继落户伊春。全市累计招商引资477亿元，实际利用外资1.83亿美元，分别比前五年增长6倍和6.5倍。对上争取成效显著，一批事关长远发展的重大政策和资金争取到位，为我市激发内生动力、助推加快发展提供了有力保障。

——过去五年，我们抢抓机遇建名城，城市功能和品位不断提升。放大棚改牵动效应，同步做大“惠民生、促城建、兴旅游、保生态”四篇文章。重大基础设施建设加速推进，路网规模持续扩大，公路等级整体提升，彻底打开了南通哈大齐工业走廊、东联煤电化基地、北接沿边开放带的大通道。林都机场通航、伊绥高速通车，飞跃式地拉近了大山与世界的距离。兴安湖水库并网发电，“三供两治”工程陆续建成投用，城市供热、供气、供水、防洪的保障能力显著提高。“四区连片”进程加快，承载功能不断增强。汤旺河等旅游名镇建设强势推进，城镇形象和服务功能大幅提升。以“西岭模式”为示范带动，梅花山、马永顺、巴兰河等特色小镇竞相涌现，“山水相依、欧风林色”的城市风格初步彰显。“四城联创”取得重大突破，创建全国文明城市获得提名资格奖；创卫获得成功，成为全省第二个国家卫生城市，开创了资源型城市和经济欠发达地区创卫的先河。

——过去五年，我们倾注全力惠民生，人民生活水平和质量大幅提高。坚持把改善民生作为一切工作的出发点和落脚点，千方百计增投入、保重点、抓落实。五年来，全市民生投入占财政总支出的比例由20%提高到60%以上，与群众利益息息相关的就业增收、住房、看病、就学等问题逐步解决。教育教学质量连年攀升，高考本科二表以上入段率由24%提高到43.1%。惠民示范区建设全面落实，覆盖城乡的医疗卫生服务体系基本形成。投入3亿多元加强社区建设，设施功能和自我管理能力显著增强。“平安伊春”建设深入推进，在全省率先实现了平安县区全覆盖。保障性安居工程全面实施，17万户、45万居民告别低矮破旧的棚户区，住进了宽敞明亮的新居，户均增加财产性收入5万元以上。在几任市委、市政府班子的积极努力下，12万老知青、大集体、“五七工”的养老问题全部解决；森工企业拖欠的工资和养老金全部补发；林企教师、公检法干警“同岗不同酬”问题彻底解决；企业退休人员养老金由月人均535元提高到1080元，林业职工工资由月人均829元提高到1917元，分别翻了一番多，林区职工群众翘首企盼“涨工资”的愿望得以实现。可以说，这五年是我市民生投入最大、人民群众得到实惠最多、各项社会事业全面进步的五年！

——过去五年，我们立足服务强素质，政府行政效能得到较大提升。创新开展了“六型”政府机关建设和“百企万民评服务”等活动，建立了与网民在线交流制度，强化了电子政务建设，健全了规范权力运行的规章制度，开通了项目审批“绿色通道”，推行了联审联办机制。主动向人大及其常委会报告工作，虚心听取政协的协商意见，认真办理人大代表意见建议和政协委员提案，自觉接受各方监督，政府机关作风和效能建设进一步加强。

此外，民族宗教、外事侨务、双拥共建、人口计生、妇女儿童、“五五”普法等各项工作都取得了新成绩。

刚刚过去的2011年，我们积极应对停伐挑战，全力以赴抓发展，经济社会与生态建设协调并进，总体向好局面得以巩固和发展。地区生产总值四个季度的增幅分别为0.2%、3.2%、6.5%和8%，正在走出停伐的阵痛期。全口径财政收入增长23%，全社会固定资产投资增长31.1%，城镇居民人均可支配收入和农民人均纯收入分别增长13.1%和16.7%。项目建设强力推进。实施重点产业项目36个，列入省推进的18个项目全面完成年度任务，西钢技改、浩水二期、友好纺纱、格润药业等项目竣工投产。产业转型升级步伐加快。蓝莓产业全方位推进，组培繁育能力达到4000万株，人工种植面积突破1万亩，加工能力达到2万吨；木制工艺品发展进入新阶段，被授予“中国木艺之乡”称号；第二届东北亚生态论坛和第十一届森林生态旅游节创新举办，首届林都伊春冰雪欢乐节隆重推出，在国际蓝莓小姐大赛、全国职工滑雪公开赛、世界六强国际女子冰壶邀请赛等系列高端赛事的推动下，冬季旅游日益火爆，旅游产业正在向四季均衡发展方向转变。招商引资实现新突破。借助哈洽会、央企对接会、香港活动周和俄罗斯“比罗比詹伊春日”等重大经贸文化交流活动，大力开展“一对一”招商，全年共引进项目157个；国内引资130亿元，实际利用外资4809万美元，同比分别增长89%和29%。城市建设全面提速。滨水工程、景观工程及高端小区等项目全面实施，省运会体育场馆及林业中心医院改扩建等公益项目加紧建设，保障性住房超额完成省下达的年度计划。中心城区和旅游名镇主街拓宽、临街建筑外立面改造同步推进，城市功能品位进一步提升。民生实事件件落实。在主渠道收入大幅减少的情况下，做到了改善民生标准不降低、推进力度不减弱，年初承诺的28件民生实事全面完成。

奋斗的历程难以忘怀，发展的经验弥足珍贵。回首过去的五年，我们深刻体会到：生态是伊春的“根”和“魂”，我们必须始终坚持生态立市不动摇；发展是解决一切问题的核心和首要，我们必须始终坚持加快发展不惜力；产业项目是牵动经济社会发展的“牛鼻子”，我们必须始终坚持大上项目不放松；执政为民是政府工作的根本宗旨，我们必须始终坚持保障和改善民生不懈怠，认认真真解民忧，实实在在帮民富，兢兢业业保民安！

各位代表，过去五年我市取得的辉煌成就，是省委、省政府和市委正确领导的结果，是市人大、市政协大力支持的结果，是全市人民同心同德、奋发进取的结果。在此，我代表市人民政府向全市各族人民，向市人大代表、政协委员，向离退休老领导、老同志，向各民主党派、工商联、人民团体和社会各界人士，向驻伊部队指战员和中省直部门，向所有参与、支持和关心伊春发展的境内外投资者、建设者和朋友们，表示衷心的感谢并致以崇高的敬意！

在看到成绩的同时，我们也清醒地认识到，过去五年的工作，很多是在补课、是在打基础、是在还历史欠账。我们经济欠发达的基本市情没有根本改变，产业支撑能力不强的现状没有根本改变，处在“不进则退、慢进也退”的现实没有根本改变。归根到底，就是林区人民群众日益增长的物质文化需求与落后生产力之间的矛盾没有根本改变。当前一切问题的根源都在于发展慢，在于政府行政效率还不高，尤其是抓发展的能力还不能完全适应当前紧迫形势的需要。对此，我们将高度重视并作为本届政府的主攻方向，全力攻坚！

二、今后五年奋斗目标和战略重点

未来五年，是伊春实现更大突破、更大发展的关键时期。我们既面临着生态保护与经济转型的双重任务，也面临着提升城市综合竞争力和提高人民生活水平的双重使命。能不能加快发展、赶超发展、跨越发展，直接关系到伊春的全面振兴，关系到全市人民的根本福祉，关系到能否与全国全省同步跨入更高层次的小康社会。市第十一次党代会对我市当前面临的形势进行了深入分析，确定了今后五至十年经济社会发展的指导思想、奋斗目标和战略举措。尽管前进的道路上挑战犹存，但我们无需畏惧！只要我们认真贯彻市委的决策部署，把全部精力集中到加快发展上来，未来的五年就一定是潜能充分释放的五年，一定是大有可为的五年，一定是跨越式发展的五年！

根据市十一次党代会精神，今后五年全市经济社会发展的总体要求是：认真贯彻落实科学发展观，高扬第三次创业旗帜，紧紧抓住国家实施天保二期工程、大小兴安岭林区生态保护与经济转型规划两大机遇，以加快发展为主题，以转变经济发展方式为主线，以改革开放为动力，以推进新型工业化、二度城市化、服务业现代化为路径，以提高人民群众生活水平为根本，加速经济建设，巩固生态建设，提升社会建设，创新文化建设，全面建设美丽富庶、文明和谐、健康幸福新伊春。

主要奋斗目标是：地区生产总值、城镇居民人均可支配收入和农民人均纯收入翻一番，财政收入和固定资产投资翻两番。实现上述目标，必须紧紧抓住以下五项战略重点：

第一，全力推动产业转型发展。按照“3+X”产业体系框架，优化产业布局，着力打造矿产开发及冶金建材、森林食品北药、森林生态旅游三大引擎产业，大力发展木材精深加工、新型装备制造、纺织、清洁能源、文化产业、现代服务业等主导产业，重点打造钢铁、钼、铅、锌、森林食品、家具、装备、纺纱8大产业链。到2016年，形成1000万吨钢铁、500万吨水泥、2.25万吨钼精粉、8万吨铅、10万吨锌、300万件家具、100万锭纱的生产能力，蓝莓种植达到20万亩以上，建成全省最大的钢铁生产和铅、锌、钼冶炼基地，全国最大的蓝莓繁育种植加工销售基地，东北三省最大的纺纱基地；家具业继续保持全国王牌地位，光明集团跻身全国同行业前列。

第二，科学构建森林经营新模式。以生态功能区建设为统领，全面实施天保二期工程，切实转变林业发展方式，通过采取全面保护、科学培育和综合经营等措施，加快森林生态系统功能的恢复。5年内，全市林木年生长量由700万立方米增加到1000万立方米以上，森林总蓄积量由2.56亿立方米增加到2.9亿立方米，整体提升对全省、全国乃至全球的生态贡献。牢固树立以市场为导向的大资源观、大市场观，深入挖掘森林生态资源的巨大潜力，让400万公顷的大森林更好地造福林区民众。

第三，全面推进城市建设提档升级。按照建设国际森林生态旅游名城的发展定位，进一步修订完善城市规划，着力构建山水园林型城市格局。投资100亿元，加快建设汤嘉铁路、伊嘉高速、嘉荫口岸、回龙湾大街延伸、伊翠铁路改造、500千伏输变电站、兴安湖水库供水、华能热电联产、体育场馆、城市污水和垃圾处理等十大基础设施工程，全面提升城市功能和承载力。强化旅游综合服务能力建设，大力发展电子商务、物流、金融以及医疗保健、文化娱乐等现代服务业，全面提高旅游城市的现代化程度。

第四，突出重点地扩大改革开放。巩固国有林权制度改革试点成果，探索形成林业多元主体经营模式。深化投融资、公益事业等重点领域、行政审批等关键环节的改革，建立更加符合市场经济规律的体制机制。以提高对外开放有效性为目的，进一步放大我市在全省沿边开发开放经济带和东北东部“12+2”市（州）合作组织中的作用；加强对俄、日、韩等国家和港澳台等地区的合作，不断提升城市国际化发展水平。

第五，持续推动民生改善。把就业和增收作为执政为民的首要任务，全力实施城乡居民收入倍增计划，尽快缩小区域、行业、群体之间的差距，走共同富裕的道路。建立收入正常增长和支付保障机制，努力实现居民收入增长与经济增长、劳动报酬增长与劳动生产率提高“两同步”。继续保持市级财政总支出60%以上用于民生的比例，不断加大与百姓息息相关的民生工程投入，让全市人民最大限度地享受到改革发展的成果。

三、2012年主要工作安排

“稳中求进”是中央今年经济工作把握的主基调。对我省而言，“稳”是大局，“进”是核心和关键。结合市情实际，我们伊春决不能安于“稳中求进”，而是要“进中求快”、“快中求好”，鼓足干劲、力争上游，多快好省地加快发展步伐，尽快做活存量，尽快做大增量，争取提前实现市十一次党代会提出的奋斗目标。这是今年乃至今后一个时期政府工作的总纲。

综合考虑各种因素，今年全市经济社会发展的主要预期目标是：地区生产总值增长12%，固定资产投资增长30%以上，规模以上工业增加值增长12%，地方财政收入增长16%，社会消费品零售总额增长15%，外贸进出口总额增长10%，城镇居民人均可支配收入和农民人均纯收入分别增长12%，城镇登记失业率控制在4.05%以内，万元GDP综合能耗下降3.5%。

为了实现上述目标，突出抓好以下10项工作：

（一）突出抓好重大项目建设。围绕重点产业发展和重大基础设施建设，加大上项目的力度，扩大投资规模。一是大举推进产业项目建设。全年计划投资50亿元，启动实施重大产业项目29个。其中，投资10亿元，续建鹿鸣钼矿采选工程，确保2013年投产；投资12亿元，建设西钢1260立方米高炉、120吨转炉和高速线材轧机生产线，年底新增100万吨钢铁产能；投资2.4亿元，续建蓝莓产业化项目，年内完成5万升红酒和30吨花青素提取生产线安装调试，酒堡营业；投资1.1亿元，续建光明创业园区，年内投产；投资1亿元，建设年产12.5万辆电动汽车总装生产线，当年开工当年样车下线；投资2.2亿元，续建 20万锭纺纱项目。二是加快推进产业园区建设。按照“林业经济林中发展、林区工业林外发展”的思路，举全市之力做大做强铁力矿产、西林冶金、伊春森林食品、光明家具、南岔建材、友好轻纺、嘉荫边贸七大产业园区，实现产业集聚发展。建立联动推进和利益共享机制，各县（市）区（局）年内至少引进并在七大园区内开工建设1个总投资超亿元的项目。对完成任务的单位除正常奖励外，给予领导班子园区建设特别奖100万元，项目达产后税收地方所得部分全部划归项目引进单位。对园区实施政策倾斜，建立特事特办的绿色通道，完善奖优促庸罚劣措施，对重大项目实施“一个项目、一套人马”的推进服务机制，重点攻坚，合力突破。三是加强重大基础设施建设。优化全市路网结构，开工建设南岔至浩良河、铁力至金山屯公路。推进中低电网改造、城市防洪工程、红山水电、红星光伏发电等项目建设，抓好500千伏输变电站立项工作。做好汤嘉铁路、伊嘉高速公路对上争取，力争尽早开工，彻底打通对俄大通道，加速推动伊春向国际化开放城市迈进。

（二）突出抓好天保二期工程。坚持不懈地保护培育森林资源，巩固扩大生态建设成果。一是全面落实森林资源管护承包责任制。按照“谁管护谁受益”和“还利于民、让利于民”的原则，对重点火险区以外的森林资源，全部以家庭为基础进行承包经营。对发展旅游、经济林和长线种植项目的承包户，遵循自然生长规律，签定长期合同，优先保证其经营权。最大限度地引入利益驱动机制，鼓励支持林地承包向大户能人集中，让承包者理直气壮获取经济利益，放大社会效益，加速推进林区生态建设。二是科学培育经营森林。把森林经营摆上突出位置，坚持育林为主，禁止变相取材，控制好抚育强度与间隔期，促进现有次生林生长，全年林木蓄积量净增700万立方米以上。大力优化树种结构，重点抓好红松、三大硬阔等苗木的人工补植，实现珍贵树种的快速增加。大力优化林分结构，充分利用森林抚育补贴试点政策，落实好316万亩抚育任务，促进林下珍贵树种更新，加速第二代阔叶红松林的建成。进一步强化森防能力建设，突出抓好林地农药、化肥的使用管理和林业有害生物防治，确保森林安全。三是实施苗木产业振兴计划。强化营林基础地位，采取苗圃改造升级、标准化建设、调整苗木培育结构、转变经营方式、建立合理定价机制和现代化营销体系等措施，逐步恢复苗圃生产能力，力争到2015年，增建3处容器育苗生产基地，保留的26处苗圃全部达到国家级标准。今年，每个林业局建设1个标准化苗圃，保证全市苗木总产量达到2亿株，成苗产量达到4000万株以上，满足生态建设和城乡绿化需求。四是强化资源综合利用。加快林场所撤并和生态移民，年内撤并28个林场所。加强林下袋栽木耳等废弃物的回收利用，减少森林污染。抓好松花江流域水污染防治规划增补项目的实施，积极推进钢铁、电力、冶金等重点节能技改项目建设，坚决完成全年节能减排目标任务。

（三）突出抓好城市建设。以筹办2014年第十三届全省运动会、举办2012年全国大学生定向越野赛和申办2014年世界大学生定向越野赛为契机，努力把城市面貌提升到一个新水平。一是大力提升中心城区功能。进一步完善中心城区总体规划，优化分工与布局。突出伊春区政治、经济、文化中心作用，强化人流、物流、资金流集散三大功能；现代服务业项目尽可能往乌马河、翠峦区摆放，工业项目向友好区集中，实施错位、互补发展。启动实施伊春河景观、沿河地质公园和步行街建设“三个一”工程，同步推进伊绥高速入口、伊春火车站广场、林都机场“三个点”标志性景观建设。加快推进文化公园、市政公园、体育公园三大主题公园建设。采取BT、BOT方式，重点抓好回龙湾大街、迎宾路、云杉路和飞龙路等9条道路建设。开工建设市客货枢纽中心。二是实施棚改异地安置工程。从发挥中心城区设施功能和减轻林业局棚改配套资金压力的需要出发，借鉴“黄金花园”和牙克石市生态移民的成功经验，出台政策措施，启动实施林区棚户区改造异地安置工程，年内在伊春区、乌马河区、翠峦区建设异地安置住宅50—80万平方米，移民1—2万人。三是加快旅游名镇和滨水城镇建设。重点抓好汤旺河省级旅游名镇和嘉荫、金山屯等市级旅游名镇建设，增强配套功能，提升旅游服务能力，力保按时完成建设任务。积极推进西岭、梅花山、达沃斯等特色旅游小镇提速扩容。抓好景观带建设，今年各区（局）址河流两岸、重点旅游景区周边、公路铁路干线两侧全部建成景观带或退耕还林还草。四是全面推进“三优”文明城市创建。建立长效机制，巩固创卫成果。扎实做好全国文明城市、森林城市、生态园林城市创建的各项基础性工作，确保早日获批。以治理脏乱差为重点，突出抓好背街小巷、居民区的污水、垃圾和冰雪处理清运工作。大力度搞好城市绿化，按照“乔灌藤草，针阔彩果”的绿化模式，调整林相结构，增加常绿树种比重，大搞平面绿化、立体绿化和垂直绿化，大造“片林”、“彩林带”和“果林带”。今年，进出各区局址2公里的道路两侧、城镇内主干道全部实施绿化，新建居民区绿化面积占总用地面积比重达到规划设计标准，新建1处以上、总面积2公顷的大型公共绿地。深入开展环境秩序综合治理行动，提高城市经营管理水平。加强文明教育，大力提升市民素质和城市文明程度。

（四）突出抓好农林产业发展。整合林果、林药、土地、林地、林木等资源要素，实施大片种植、大片培育、基地化生产、链条式加工，不断提高产品附加值和市场竞争力。一是做强森林食品北药业。全力打造蓝莓、食用菌、北药三大产业链。培植壮大升辉、忠芝、双蓝等龙头加工企业，同步推进蓝莓组培繁育、种植、深加工和销售网络建设，尽快形成从初级生产向高端加工的完整产业链条。新增蓝莓种植面积2万亩，加工能力达到5万吨。培植壮大林都、兴安塔等龙头企业，重点在引进战略投资者、专业大户和龙头加工企业上下功夫，尽快提高黑木耳、香菇、猴头蘑等产品的加工转化能力，食用菌规模达到6.2亿袋，加工能力达到7000吨。培植壮大葵花、格润、祥峰等龙头企业，带动北药基地加快发展，种植面积达到15.7万亩，林下药材改培面积达到44万亩。积极推进蓝靛果、猕猴桃、树莓等山野果的改培和产品开发，重点抓好乌伊岭万亩榛子林改培项目，争取尽快形成新的产业链条。抓好冷链仓储物流业，淡吞旺吐，保障龙头企业原料供应，增加群众采山和种植收入。二是集约化发展生态畜牧业。以建设全省优质安全畜产品供应基地为目标，以解决“节本、提质和增效”三大难题为重点，稳步推进常规养殖，突出发展特色养殖，着力提高产业化水平。年内生猪、奶牛、野猪和禽类饲养量分别达到80万头、4.5万头、3.5万头和2500万羽。三是全力推进农业产业化。以调优种植结构为重点，年内水稻、玉米面积扩大到60万亩和75万亩。重点抓好铁力市200吨水稻智能化催芽育秧基地建设，落实国家粮食高产创建项目。加快灌区、节水改造、病险水库加固等农田水利设施建设。大力推进森工多种经营用地流转，将分散的土地向国营农场、大型农机合作社和种植大户集中，鼓励跨区域发展农机农技合作社，提高土地产出能力，增加林业务农职工收入。重点抓好双丰、铁力、桃山集中连片多种经营用地的流转工作。年内建成大型现代农机合作社2个，新建农民和林业务农职工专业合作社50个。同时，巩固扩大局场县共建成果，全面提升新农村、新林区共建层次。四是开展生物质能源试点。充分利用枝桠材、林间可用剩余物以及平榛等速生植物，大力发展生物质能源。年内在南、北、中部建设3个试点，取得经验后，全面推开。五是整合做强林产工业。采取政府统筹、市场化运作的办法，将36万立方米抚育伐出材和境外进口木材向龙头企业集中，借停伐之机把林产工业企业整合到位。人造板以龙乡、大福为龙头，协调原料等生产要素，保证满负荷生产，并积极寻求合作企业，加快推动产业升级。家具以光明、双丰、友春为龙头，大力实施品牌战略，推动上下游企业联名联牌、链条式发展。木制工艺品以侨艺、亿通为龙头，整合企业工艺优势，在挖掘文化内涵，拓展产品利润空间上求突破，把小产品打造成大产业，加速叫响“中国木艺之乡”品牌。

（五）突出抓好旅游业转型升级。以启动实施“全国森林生态旅游示范区试点”为牵动，加速推动四季旅游均衡发展。一是全力推进景区和基础设施升级。重点改造提升汤旺河国家公园等21个A级以上景区、桃山等3个国家级狩猎场、梅花山等4个S级以上滑雪场、大丰河等8个省级达标漂流河段。投资6.4亿元，开工建设美溪全国总工会疗养度假中心；投资1.5亿元，开工建设乌马河南沟高端旅游度假区。抓好桃山旅游综合开发项目前期工作，尽快启动实施。加大对已开工星级酒店的推进力度，确保按签约时限完成建设任务。二是全力推进产品设计和展会策划升级。坚持以游客为本，人性化设计旅游指南，强化伊春旅游网建设，方便和满足游客需求。多视角开发四季旅游产品，重点抓好温泉、山泉养生药浴、雪山穿越、林海人家体验等特色产品开发，做大叫响林都伊春森林冰雪欢乐节、汤旺河冰雪龙舟赛等原创品牌。探索运用市场化手段，引进品牌策划组织、广告商和战略投资者参与举办重大节会和赛事，减少政府投入，实现共赢发展。三是全力推进接待服务升级。重点提升“地接”能力，突出抓好中心城区、铁力市、嘉荫县、金山屯区“一主三副”4个旅游集散地建设。加强与中青社、国旅等品牌旅行社合作，整合做强一批市内旅行社，国际化、市场化地搞好旅游推介营销。全年旅游收入增长23%以上。

（六）突出抓好体制机制创新。在继续推进各项改革的基础上，以增强造血功能为目的，重点推进投融资体制改革。启动实施1个试点，做大3个融资平台，建立1家伊春人民自己的银行。启动林业碳汇交易试点。借鉴义乌经验，加大向国家林业局争取力度，尽快开展第一笔碳交易。做大城市资本运作平台。将水解厂等破产企业回购资产、大连光明四户回购企业的优良资产以及兴安湖水库资产注入城投公司，把现金流做大，提高融资能力，服务于城市建设，并为未来上市夯实基础。做大旅游资本运作平台。组建汤旺河国家公园旅游股份公司，政府以支付过桥资金的方式与企业共同搭建平台，向社会募集资金，以股权融资等形式吸引投资，循序渐进地将全市优良旅游资源整合进来，努力做成旅游航母。做大林业资本运作平台。在搞好森林认证和森林湿地价值评估的基础上，通过分类整合，搭建平台，将可运作的资产剥离出来，引进战略投资者进行资本化运营，把未来的“绿色储蓄”变成可以用于生态保护和经济转型的资金。成立伊春市农商行。加速推进农信社改制，争取伊春农商行尽快挂牌并投入运营，为全市中小企业发展提供强有力的资金支持，解决“融资难”问题。

（七）突出抓好招商引资和对外开放。充分利用“两种资源”、“两个市场”，大力提升对内对外开放水平。一是全力谋划招引产业项目。围绕拉长产业链条、做大产业园区谋划项目，分类落实引资任务，提高招商引资的针对性和有效性。今年产业项目招商重点围绕打造“三城一都”这个主攻方向，组织专业队伍实施“一对一”、“点对点”招商。“铁力钼城”重点围绕钼铁、钼酸铵、钼圆片、钼金属超细粉喷涂四大主产品、硫酸等副产品、球磨机衬板和钢球等选矿配套产品产业链抓招商。“西林钢城”重点围绕矿山开发以及钢材、铅锌等上下游产业链抓招商。全力支持西钢获得翠宏山、翠东铁矿采矿权。成立伊春矿产资源勘探公司，做好地质勘查和深部、边部找矿，把未来矿山开发主动权紧紧抓在政府手里，让大自然赐予我们的宝贵资源发挥出最大效益。“山果酒城”依托中心城区已经形成的蓝莓等果酒和地方土酒的品牌优势，谋划一批养生保健酒、高档礼品酒、纪念酒等项目，系列化、品牌化抓招商。“森林食品之都”重点围绕全市食用菌、北药深度开发谋划生成项目，打捆对上争取和招商引资，尽快形成集聚发展之势。全年引进到位资金200亿元。二是突出重点地开拓国内国际市场。强化与东北东部“12+2”市（州）区域合作，积极组织参加“哈洽会”等重大展会活动，强化务实合作。巩固欧美、东南亚等传统市场，加强与日、韩的联系合作，办好“韩国伊春周”，努力扩大合作领域和贸易规模。出台促进外向型经济发展的政策措施，鼓励支持各类企业开展“两头在外”和流通型贸易，扩大产品出口比重。三是深化对俄经贸合作。全力抓好嘉荫口岸固冰浮箱通道、通关公路、比罗比詹伊春展销中心、帕什科沃木材工业园以及嘉荫蔬菜出口基地建设。办好“伊春比罗比詹日”，进一步拓展交流合作领域。支持乌马河、上甘岭等对俄劳务输出企业加快发展，今年在俄采伐木材80万立方米以上。

（八）突出抓好科技和人才工作。大力实施创新驱动和人才引领战略，让科技和人才成为撬动经济发展的有力杠杆。一是加强科技创新体系建设。发挥市林科院和带岭林科所等科研机构的作用，加速建立完善以森林食品为代表的产业技术创新战略联盟。围绕种苗、林果等林业资源开发，重点实施蓝莓培育与精深加工、名贵山禽及冷水鱼规模化养殖等市级以上科技项目20项。强化知识产权保护，突出抓好蓝莓、红松籽、平贝等独具小兴安岭特色的稀有产品技术标准的制定和申报，争取开发销售主动权。二是加快推进科技成果转化。搞好科研项目与生产领域的对接，孵化培育一批高新技术和创新型企业。加强科学知识传播体系建设，深入推进全民科学素质纲要的贯彻落实，突出抓好科技新成果在林下经济、特色经济中的推广应用。今年全市应用类科技成果应用率达到80%以上。三是积极开展“府校共建”。吸引国内外著名学府和科研院所在我市设立科研工作站、研发中心、产业科研基地等机构，重点建好中国人民大学伊春生态学院、林管局和林科院博士后科技工作站、生态监测站、院士工作站等“一院四站”，争取引进苏宁集团云计算项目，集聚高端人才、高端技术推动伊春发展。四是完善人才引进使用政策。开辟高端和急需人才引进的“绿色通道”，放宽文化、教育、体育、园林、木艺、酒艺等领域人才引进自主权。建设“中小企业创业园”和“大学生创业园”，为人才成长搭建平台。设立科技创新奖励基金，重奖有突出贡献的人才。兑现国家和省人才政策待遇。

（九）突出抓好以改善民生为重点的社会建设。进一步加大投入，全面发展社会事业，让全市人民共享发展成果。一是全面实施“6541”文化工程。重点抓好带岭影视城、梅花山文化基地、乌马河画家村、图书馆、马永顺纪念馆、小兴安岭美术馆、大森林音乐厅等文化项目建设。免费向市民开放恐龙博物馆、伊春森林博物馆以及各类图书馆、文化馆。二是做强做优教育事业。加大教育投入，改善办学条件，提高教育教学质量。抓好公办幼儿园建设，普及学前3年教育；整合教育资源，加速推进义务教育均衡发展；巩固优质普通高中教育，做优做强市一中，打造品牌名校；加快发展职业教育，抓好市实训基地建设，支持伊春林校创建“国家中等职业教育改革发展示范学校”，加快推进职业学院专升本。三是提升体育事业战略地位。全面进入筹办第十三届全省运动会的备战状态，加快体育场馆、训练基地建设和公共体育设施建设。深入开展全民健身活动，增强市民体质。继续办好全国职工滑雪公开赛、国际冰壶邀请赛等品牌赛事，促进体育事业与旅游产业的有机融合。四是强化公共卫生服务体系建设。进一步完善覆盖城乡的公共卫生医疗服务体系，发挥林场所、农村和社区卫生服务网络作用，满足人民群众就医需求。抓好全市突发公共卫生应急指挥体系建设。加快发展康体养生产业，吸引国内外著名医院到我市建立康复中心。五是努力扩大就业。认真落实就业政策，加大对新增失业人员就业援助力度，多渠道开发就业岗位，全年新增就业3.5万人。六是提高社会保障能力。扩大城镇职工基本养老保险等各类保险的覆盖面，强化基金征缴和管理，健全城乡居民低保与物价变动相适应的动态调整机制。加强社会救灾救济和“保供稳价”工作，保障困难群众的基本生活。七是加强和创新社会管理。高度重视停伐转型期出现的社会矛盾和问题，强化社会治安综合管理，深入开展“平安伊春”建设。加强安全生产监管，全面落实企业主体责任，加大事故隐患排查和治理力度，有效防范各类安全生产事故的发生。认真做好信访工作，深入开展矛盾纠纷排查和调处工作，以高度负责的精神对待人民群众的诉求，理顺情绪，化解矛盾。继续做好民族宗教、国防动员、外事侨务、双拥共建、人口计生等各项工作。

今年，市财政用于民生方面的专项支出同比增长20%以上，重点办好17件民生实事：

1、继续实施保障性安居工程，新建改建住房5.5万户、300万平方米，改善10万群众的居住条件。

2、实施换热站及供热一级网改造升级工程，提高供热能力，满足群众住“暖屋子”的诉求。启动华能集团投资30亿元、建设两组35万千瓦机组的热电联产项目，力争用2—3年时间，彻底改善中心城区供热条件。

3、实施供水项目27个，自来水普及率同比提高3个百分点，达到70%。解决中心城区部分居民小区分段定时供水问题。启动实施兴安湖水库供水工程项目，建设中心城区第二水源地，全面提高供水保障能力。

4、启动实施污水和垃圾处理“打捆”工程，用2—3年时间改善各区（局）排污问题，让河流更清澈、环境更优美。

5、为每个县（市）区（局）配备1台（套）现代化清雪设备，降低环卫工人劳动强度，确保群众冬季出行安全。

6、为每个县（市）区（局）和大型宾馆配备1台（套）国际最先进的体检设备，为老百姓和游客提供方便快捷的医疗服务。

7、启动伊春林业中心医院三级甲等医院创建工作，努力为林区群众创造更加优良的就医条件。

8、改造建设两家大型现代社区综合服务中心，进一步增强社区综合服务能力，更好地服务社区百姓。

9、在伊春、新青和南岔区各改造建设一个容纳500人以上的标准化养老院，提高为老服务能力。

10、在中心城区建设以体育馆、体育场为主的竞技体育公园，改造建设开放式的休闲健身体育公园；改造升级100个全民健身场地设施，满足建设“健康伊春”的需要。

11、新建5所公办幼儿园、2所乡镇中心园，实现每个区（局）至少有一所以上公办幼儿园的目标，让每个适龄孩子都能入园。

12、全面开展学校“五安”工程建设，对饮食、消防、治安、交通和校车安全进行系统整治并建立长效机制，从源头上杜绝各类安全事故，让家长放心。

13、强化寄宿制食堂建设，市财政安排专项资金，县（市）区（局）适当配比，对全市寄宿制学校食堂给予补贴，一律不准对外承包；加大对校内食堂和校外“小饭桌”的监管，让孩子们一日三餐吃得更好。

14、继续实施人口计生“惠家工程”，兑现当年城乡独生子女父母奖励费，决不出现新的拖欠，并逐步偿还历史陈欠。

15、加强食品药品质量监督和价格监管，严厉打击制假售假和价格欺骗等违法行为，让群众吃上健康安全的食品药品；落实基本药物零差价销售制度，着力解决“看病贵”和“以药养医”等问题。

16、开发公益性岗位2000个，为特殊群体提供就业空间，改善生活条件。

17、投入扶贫创业资金500万元，建立扶贫创业基地50个，提供扶贫创业岗位2000个，助学1000名，让困难群众生活得更幸福、更有尊严。

（十）突出抓好政府自身建设。今年是新一届政府工作的开局之年，面对新的形势和任务，市政府将牢记使命，履职尽职，树立新形象，展示新作为。一是大力提高行政效能。以“六型”政府建设为统领，以实施“十大绩效工程”为抓手，对市党代会确定的战略任务、市委经济工作会议部署的“1031”工程以及《政府工作报告》安排的各项工作进行合并分解，形成10个板块的绩效工程实施方案，每个副市长（副管局长）负责一个绩效工程，一个本子抓落实。科学制定考评办法，邀请市人大代表、政协委员评议监督，一套评议督到底，确保各项决策部署落到实处。二是切实推进依法行政。健全政府决策机制，扎实推进政务公开，深化电子政务建设，规范行政执法行为，进一步落实行政执法责任制。自觉接受人大及其常委会的法律监督和工作监督，大力支持政协履行政治协商、民主监督和参政议政职能。认真办好人大代表的议案、建议、批评、意见和政协委员提案。广泛听取民主党派、工商联、无党派人士和各人民团体的意见，高度重视司法监督、舆论监督和社会公众监督。三是加强廉政建设。坚持从严治政、科学管理，认真落实党风廉政建设责任制，加强以完善惩治和预防腐败体系为重点的反腐倡廉建设。发挥公共资源交易中心作用，完善部门预算、政府采购、项目公开招投标等制度，加强对天保等专项资金、重大投资项目、债券使用和权力部门的审计监察，严肃查处各类违法违纪行为，坚决纠正损害群众利益的不正之风。对影响经济发展的“中梗阻、底梗阻”行为严查重处，决不姑息。收紧财政盘子，狠抓增收节支，降低行政成本。今年，各种政府会议、文件同比压缩20%，公车配置、公费出国（境）和公务消费同比压缩20%，把省下来的钱用到促发展、惠民生上来。

各位代表，同志们！过去的五年，我们谱写了伊春发展史上辉煌璀璨的新篇章；未来五年，我们承载着建设新家园、实现新跨越的光荣使命。让我们在市委的正确领导下，承前启后，继往开来，同心同德，创新创业，向着建设美丽富庶、文明和谐、健康幸福新伊春的宏伟目标阔步前进！
